# Uncommon Presentations of Nasopharyngeal Carcinoma: A Report of Two Cases

**DOI:** 10.7759/cureus.69643

**Published:** 2024-09-18

**Authors:** Alekhya Vemula, Shanthi Priya Dhanasekaran

**Affiliations:** 1 Otolaryngology, Head and Neck Surgery, Saveetha Medical College and Hospitals, Saveetha Institute of Medical and Technical Sciences, Saveetha University, Chennai, IND

**Keywords:** atypical presentation, chemotherapy, chronic headache, nasopharyngeal carcinoma, radiotherapy

## Abstract

Nasopharyngeal carcinoma (NPC), a rare form of squamous cell carcinoma originating from the nasopharynx epithelium, exhibits a higher prevalence in southern China, Southeast Asia, the Arctic, North Africa, and the Middle East, with significant incidence in northeastern India, particularly Nagaland. Commonly presenting with nasal and otological symptoms, NPC diagnosis is challenging due to its diverse clinical manifestations. This case report highlights two atypical NPC cases: a 32-year-old female presenting with chronic headache and giddiness and a 22-year-old male with severe right-sided facial pain and trismus. Both cases underwent extensive diagnostic procedures, including imaging and biopsies, ultimately confirming NPC. Treatment involved radiotherapy and chemotherapy, resulting in significant symptom improvement. These cases underscore the importance of recognizing unusual NPC presentations to facilitate early diagnosis and treatment, improving patient outcomes.

## Introduction

Nasopharyngeal carcinoma (NPC), once termed lymphoepithelioma, is a form of squamous cell carcinoma that develops from the epithelial tissue of the nasopharynx [1]. It is an uncommon cancer in most parts of the world, with age-standardized incidence rates of less than one per 100,000 person-years, with ratios of 0.6-2.0 per 100,000 males and 0.2-0.8 per 100,000 females globally [2]. Significantly higher rates were noted in the Cantonese population of southern China, intermediate rates among indigenous groups in Southeast Asia, the Arctic, North Africa, and the Middle East. The National Cancer Registry has reported that NPC accounts for 1.82% of all cancers in the northeastern states of India, making it the eighth most common cancer in the region. Nagaland has the highest incidence, at approximately 4.3 per 100,000. The age-adjusted rate of NPC in Kohima district, Nagaland, is 19.4 per 100,000, one of the highest recorded rates [3].

The 1978 World Health Organization (WHO) classified NPC into three main histopathological sub-groups: keratinizing differentiated type (20-25%), non-keratinizing differentiated type (10-15%), and non-keratinizing undifferentiated type (60-65%) [4]. In 1991, the classification of NPC was modified into three categories: keratinizing squamous cell carcinoma, non-keratinizing carcinoma, and basaloid carcinoma. Non-keratinizing carcinoma is further divided into differentiated and undifferentiated subtypes where the proportion of keratinizing squamous cell carcinoma among all NPCs is likely higher in low-incidence areas compared to high-incidence areas. The most common type is the undifferentiated non-keratinizing type which is strongly associated with Epstein-bar virus infection.

It is said that 80% of NPC patients commonly present with nasal symptoms like nasal obstruction, bleeding, or postnasal drip. About 50% of NPC patients experience otologic issues like unilateral hearing loss or tinnitus resulting from middle ear effusion [1]. Neck masses and cranial nerve (CN) palsies are other known common presentations of NPC. This case report is significant for highlighting the uncommon presentations of NPC. In both cases, the patients did not exhibit the classical symptoms of NPC in the early stages, leading to delayed diagnoses. Consequently, both patients presented with advanced-stage disease with overlapping symptoms. This report underscores the challenges in early detection and offers insights from the literature on strategies that could be implemented to improve early diagnosis rates in cases of mild suspicion of NPC.

## Case presentation

Case 1

A 32-year-old female presented to the emergency department with complaints of giddiness and vomiting for the past two days. She had a history of chronic headaches for the last four months, throbbing type in the bilateral temporal region, with no known aggravating factors, and had temporary relief with medication. She had no known comorbidities and had no significant medical or family history. The patient reported a prior consultation for headache four months back when a computed tomography (CT) brain and refractive error check was done, and both of them were normal. She was then treated for migraine and had been prescribed analgesics, and anti-migraine medications. There was no history of nasal blockage, discharge, postnasal drip, facial heaviness, hearing loss, tinnitus, neck pain, photophobia, or blurring of vision.

Upon admission, a repeat CT brain was done in the emergency department, which was normal. An otorhinolaryngologist consultation was sought to investigate potential vestibular cause for giddiness. Physical and neck examination was normal, with no palpable lymph nodes. Dix-Hallpike maneuver and Romberg’s test were negative, and no limb weakness was noted. A provisional diagnosis of migrainous vertigo was made, and the patient was started on cinnarizine, betahistine, and analgesics for symptomatic management. The following day, she had symptomatic improvement but was not fully recovered.

**Figure 1:**
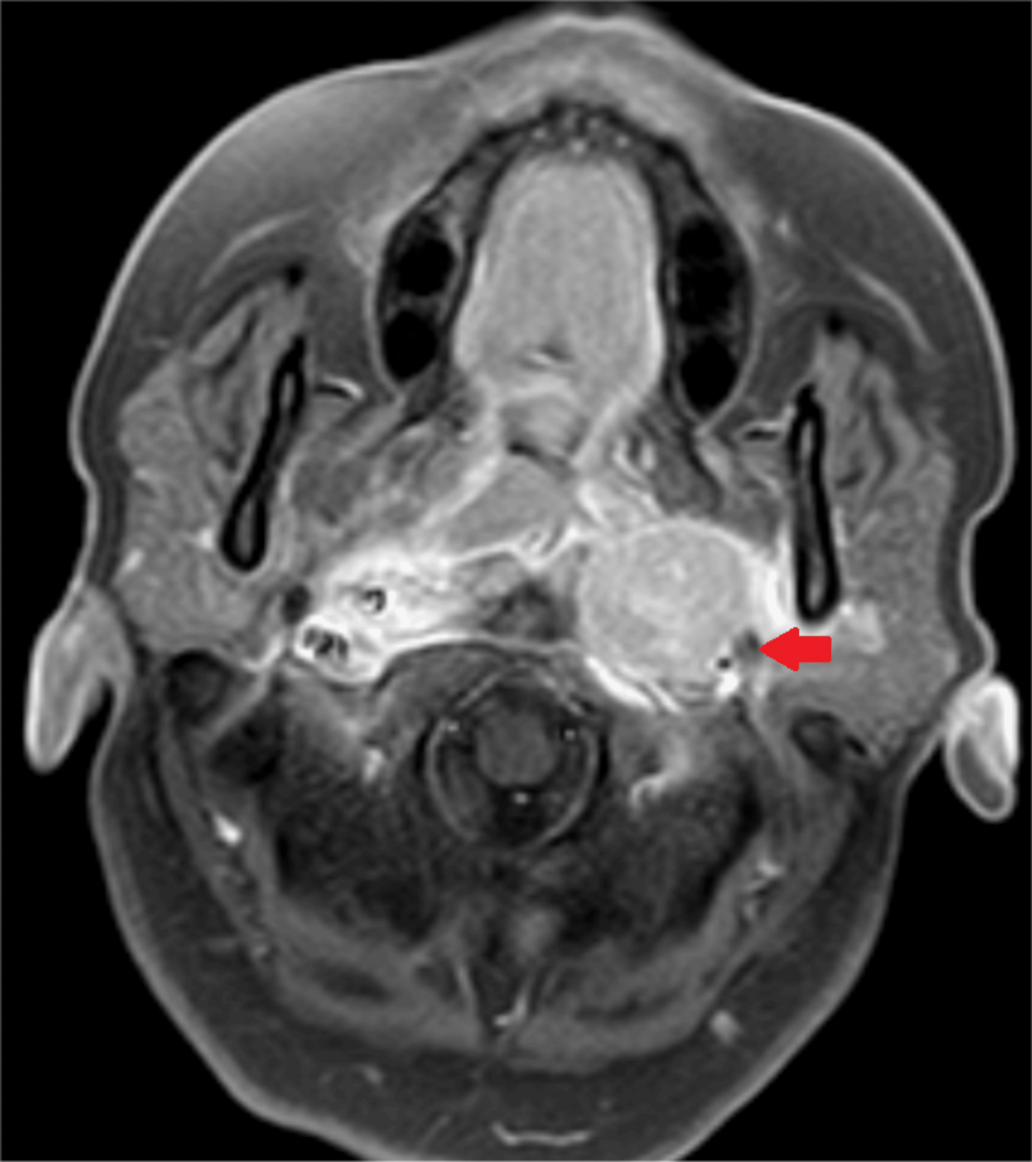
Contrast-enhanced MRI brain with inner ear Case 1: This axial section shows a hyperintense mass lesion in the nasopharynx (red arrow) that extends along the posterior nasopharyngeal wall and involves pharyngeal mucosal space with adjacent similar homogenous enhancing nodal masses in bilateral parapharyngeal space. MRI, magnetic resonance imaging

Pure tone audiometry with impedance testing showed a bilateral Ad-type curve with a hearing threshold of 13.3 decibels (dB) on the right side and 18.3 dB on the left side suggesting a bilateral hearing threshold within normal limits. Vestibular-evoked myogenic potential (VEMP) testing indicated bilateral P13 and N23 mild prolonged latencies, suggesting mild inferior saccular defect. For further evaluation, a contrast-enhanced magnetic resonance imaging (MRI) of the brain and inner ear was made, which revealed a well-defined homogeneous T1 iso-intense, T2 hyper intense 3x2.5x2.1 centimeter (cm) soft tissue lesion along the posterior nasopharyngeal wall involving the pharyngeal mucosal space (Figure 1). Similar homogeneously enhancing nodal masses were seen in the bilateral para-pharyngeal space and level 2 regions.

**Figure 2:**
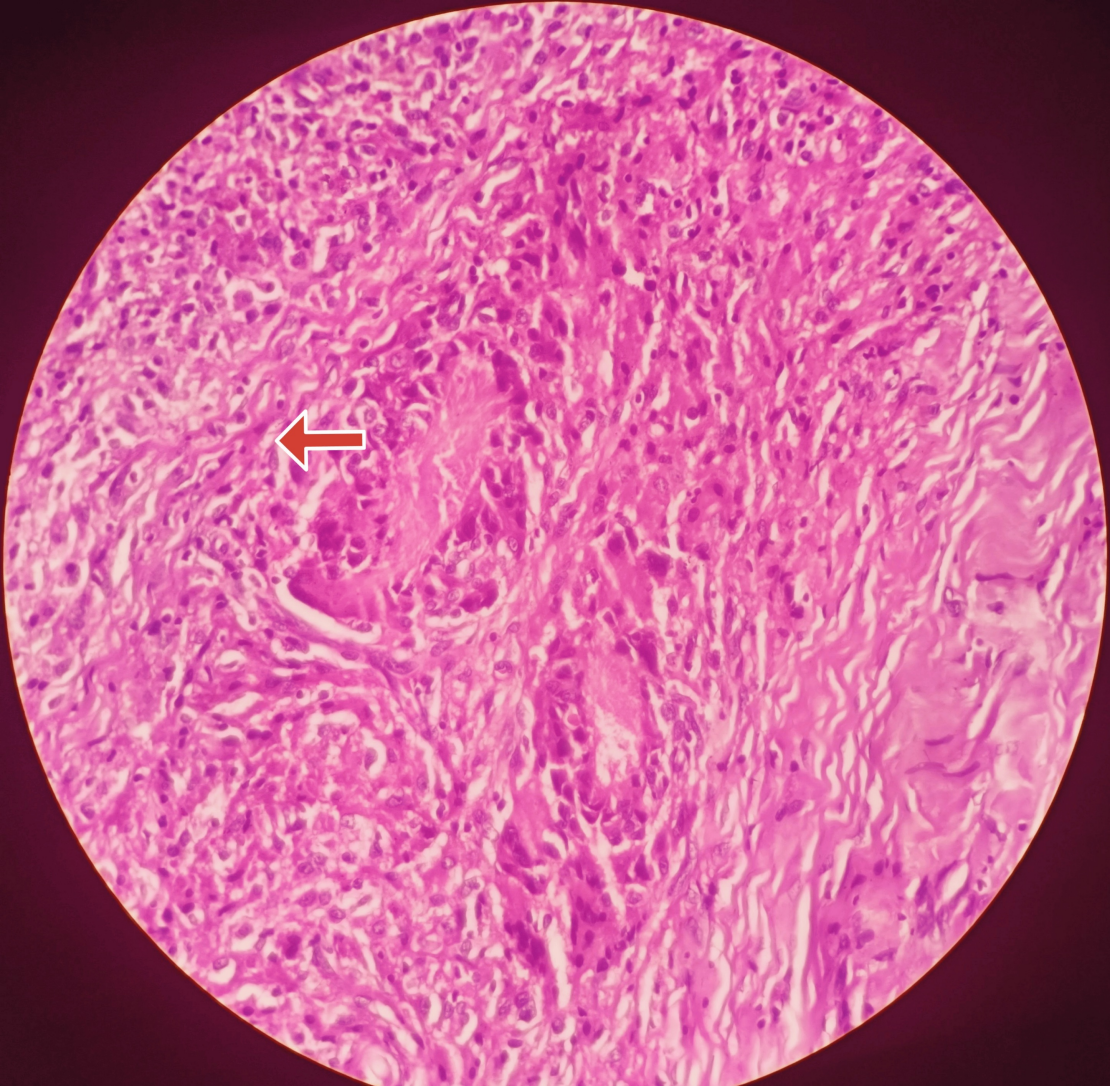
Histopathology of nasopharyngeal biopsy shows non-keratinizing undifferentiated NPC Case 1: The tumor cells are arranged in syncytial and trabecular patterns. The cells are seen invading the stroma (red arrow). The cells have moderate eosinophilic to amphophilic cytoplasm, round nuclei with vesicular chromatin in the background of lymphocytes, and plasma cells with no evidence of keratin. NPC, nasopharyngeal carcinoma

Then a diagnostic nasal endoscopy was done, which revealed a proliferative mass in the left side of the nasopharynx extending toward the right side but not into the nasal cavity. In suspicion of malignancy, ultrasound-guided fine needle aspiration cytology (FNAC) of the left level 2 lymph node was done, which showed metastatic carcinomatous deposits. An endoscopic biopsy of the nasopharyngeal mass under general anesthesia and histopathologic examination confirmed undifferentiated non-keratinizing NPC (Figure 2). A whole-body positron emission tomography (PET) scan was done to assess distant metastasis and the tumor was staged as T3N2M0. The patient underwent intensity-modulated radiotherapy (IMRT) to the primary tumor and lymph nodes at a dosage of 70 gray in 35 fractions for seven weeks along with three cycles of concurrent chemotherapy with cisplatin 75mg/m^2^ as suggested by the medical oncologist resulting in complete resolution of giddiness and headache, with no recurrence of symptoms for six months before losing follow-up.

Case 2

A 22-year-old male came with complaints of right-sided temporal headache for the past two years, which was aggravated for the past six months. The headache was throbbing, continuous in nature radiating to the right side of the face, relieved slightly on taking analgesics, and recurred later. There was a history of restricted and painful mouth opening for six months, which was insidious in onset and gradually progressive in nature. No history of nasal block, nasal discharge, postnasal drip, epistaxis, hard of hearing, tinnitus, vertigo, dysphagia, and voice change. No history of diplopia, blurring of vision, neck stiffness, neck rigidity, nausea, and vomiting was present either. He was a chronic smoker with no known co-morbidities. Medical and family history was insignificant. On physical examination, the right tympanic membrane was congested and retracted. Mouth opening was restricted to less than two finger breadth. Examination of the neck revealed firm swelling extending along the right parotid and right temporomandibular joint region, which was tender on palpation; no warmth was noted. The right parotid appeared bulky and the right level 2 lymph node was palpable, hard in consistency, and immobile with no tenderness or warmth.

Previously patient had a consultation outside in view of the restricted mouth opening where an MRI temporomandibular joint was done, which showed a right para-pharyngeal mass with mandibular nerve infiltration. Video-laryngoscopy showed normal findings with mouth opening restricted. On diagnostic nasal endoscopy, a smooth bulge was noted in the right lateral wall of the nasopharynx. No proliferative growth was visualized.

**Figure 3:**
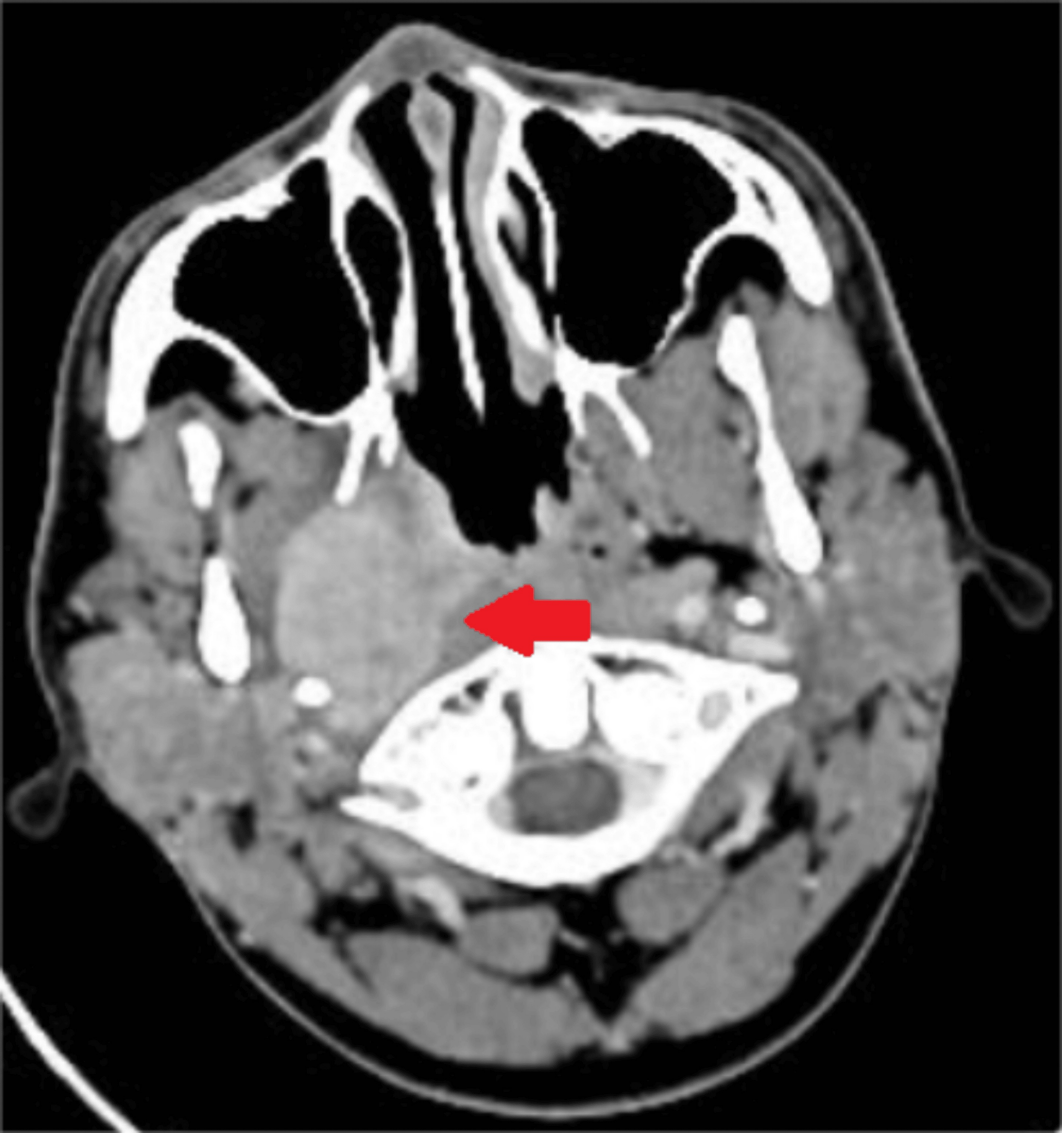
Contrast-enhanced CT neck Case 2: This axial section shows a hyperdense mass lesion in the right para-pharyngeal space and the right side of the nasopharynx (red arrow). The mass lesion involves the right lateral wall of the nasopharynx, obliterating the fossa of Rosenmuller and torus tubaris. Posteriorly displacing the carotid and internal jugular vessels, extends posteromedially to the prevertebral space, abutting the right prevertebral muscles and posterolaterally related to the deep lobe of the parotid gland. CT, computed tomography

Contrast-enhanced CT of the skull base to thorax was done, which showed an ill-defined 5x4.8x6 cm, heterogeneous mass lesion epi-centered in the right para-pharyngeal space, extending from the skull base to the C2 vertebra. The mass was seen to involve the right lateral wall of the nasopharynx, obliterating the fossa of rosenmuller and torus tubaris (Figure 3). Anterolaterally, it was related to the right lateral pterygoid muscle, posteriorly displacing the carotid and internal jugular vein. Superiorly, it was related to the petrous segment of the right internal carotid artery and foramen ovale, with attenuation of the petrous, lacerum, and cavernous segments and was seen to extend posteromedially to the prevertebral space, abutting the right prevertebral muscles, and posterolaterally, was related to the deep lobe of the parotid gland and styloid process. Prominent heterogeneously enhancing lymph nodes were noted in the right level 2, posterior triangle level 5, and right intra-parotid gland, with the largest being 2x1.8 cm in the right level 2 station.

In suspicion of malignancy, ultrasound-guided FNAC was done from the largest level 2 station node, which showed occasional clusters of spindle cells with few inflammatory cells in a background of hemorrhage with features suggestive of spindle cell lesion. The patient underwent a nasopharyngeal biopsy under general anesthesia, which showed inconclusive results. Further ultrasound-guided FNAC was done from the right intra-parotid lesion, which only showed lymphoid cells with occasional epithelial cells without any atypia.

**Figure 4:**
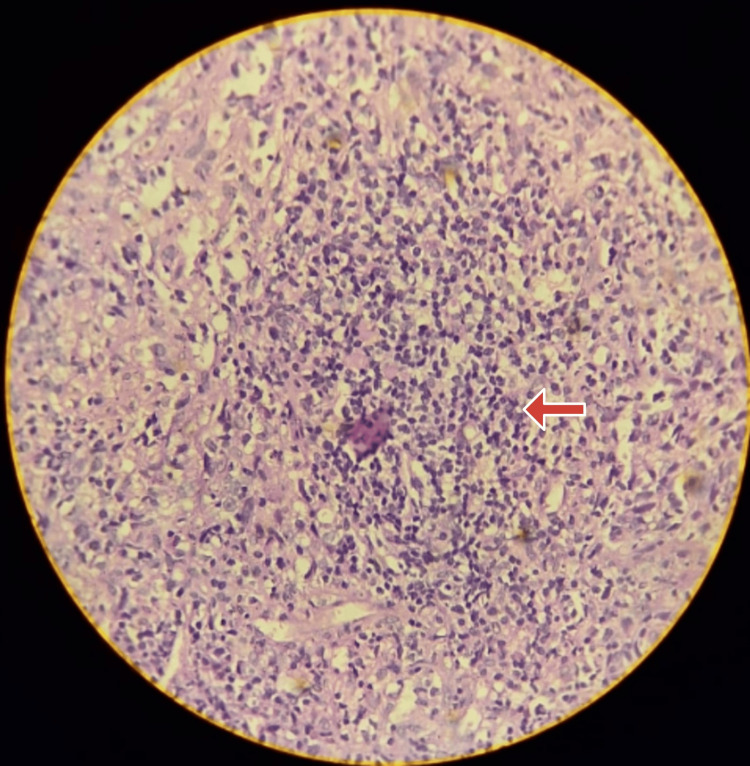
Histopathology of nasopharyngeal biopsy shows non-keratinizing undifferentiated NPC Case 2: Tumor cells arranged in irregular sheets (red arrow) with moderate eosinophilic cytoplasm and moderately pleomorphic irregular vesicular nuclei with prominent nucleoli with occasional cells showing mitoses. Occasional bizarre cells are also seen. These cells are surrounded and overlapped by dense sheets of lymphocytes. NPC, nasopharyngeal carcinoma

The patient was then discharged at request and upon review, an ultrasound-guided biopsy was done from the right level 5 lymph node, which was not palpable manually. Immunohistochemistry showed strong membranous positivity for pan-cytokeratin in 90% of cells, CD45 negative, and P63 strong to moderate nuclear positivity in 90% of the cells suggestive of metastatic carcinomatous deposits. Since the para-pharyngeal lesion was inaccessible for image-guided biopsy, a repeat biopsy from the right side of the nasopharynx was taken assuming that inconclusive results were due to sampling error. Histopathology was then reported as a non-keratinizing poorly differentiated carcinoma (Figure 4) of the nasopharynx with right para-pharyngeal extension. A whole-body PET scan was done to assess distant metastasis and the tumor was staged T4N2M0.

The patient then underwent three cycles of neo-adjuvant chemotherapy with cisplatin 80 mg/m^2^ and gemcitabine 1 gm/m^2^ and is currently planned to undergo concurrent chemoradiotherapy (CTRT) with cisplatin 100 mg/m^2^ planned to be given on day one of week one, four, and seven for seven weeks along with delivering radiotherapy with a total dose of 70 gray in 35 fractions. On follow-up, his mouth opening completely improved, pain improved significantly, and the size of the swelling on the right side of his neck decreased. Although the patient had side effects of chemotherapy, tolerability to pain improved significantly.

## Discussion

In the first case, the patient presented with giddiness due to eighth nerve involvement, which is uncommon in NPC. The findings of VEMP indicating a mild infarct in the inferior vestibulocochlear pathway, absence of giddiness after radiotherapy, and absence of compression on the brainstem, which could impact central balance regulation, support that the giddiness might be linked to the involvement of the eighth CN. Clinical examination of the trigeminal nerve was normal. Although radiologically, there was no clear evidence of trigeminal nerve involvement, the mass compressing the carotid spaces and displacing the pterygoid muscles raises suspicion of possible V3 nerve infiltration, which might cause temporal headaches either through referred pain or by affecting the branches supplying the temporalis muscle. This case highlights a thorough diagnostic approach: initial normal CT brain results followed by targeted otorhinolaryngology and vestibular evaluations, re-evaluation, and advanced imaging, which led to the accurate diagnosis of T3N2M0 NPC, demonstrating the effectiveness of the diagnostic strategy. Complete symptom resolution and six-month recurrence-free follow-up underscore the effectiveness of the treatment and its potential application for similar cases. This also provides valuable insights into NPC with nonspecific symptoms like giddiness and headache, which suggest considering malignancy in the differential diagnosis, especially when persistent symptoms cannot be explained by other causes, even in the absence of classic signs. Although the six-month follow-up demonstrates promising short-term outcomes, a longer follow-up would provide further insights into recurrence risk and long-term efficacy. Additionally, exploring the rationale behind selecting IMRT and chemotherapy over alternative treatments could enhance the educational value of the report.

In the second case, difficulties in performing the initial biopsy were encountered due to restricted mouth and difficult orotracheal intubation. MRI of the temporomandibular joint initially identified a right parapharyngeal mass with infiltration of the mandibular branch of the trigeminal nerve. Subsequent contrast-enhanced CT of the skull base revealed that mass extending into the lateral pterygoid muscle, involving the foramen ovale and foramen lacerum, is indicative of possible perineural spread along the trigeminal nerve. Involvement of the mandibular nerve along with irritation or compression of adjacent masticatory muscles and pain from trigeminal neuralgia may contribute to trismus by inducing muscle spasms and causing the patient to limit jaw movements to avoid exacerbating painful episodes. Despite the initial biopsy result being inconclusive, the team’s persistence in performing additional biopsies, including a repeat nasopharyngeal biopsy and ultrasound-guided biopsy from a non-palpable lymph node, ultimately led to a definitive diagnosis. Once diagnosed, the patient’s concurrent CTRT led to significant improvements in pain, mouth opening, and neck swelling after the first cycle, indicating that early treatment can be effective even in advanced cases. However, overlapping symptoms led to delays in diagnosing NPC, which only became clear after multiple inconclusive biopsies. Although a frozen section is not routinely advocated in the nasopharyngeal biopsy, an on-table frozen section would have been helpful in this case as a definite proliferative lesion was not visible on endoscopy. Apart from this, the patient’s financial limitations and intolerance to pain contributed to delays in evaluation and treatment, affecting the overall timeline of care. Neither Epstein-Barr virus (EBV) serology testing nor EBV latent membrane protein (LMP) immunohistochemistry was done in both cases as it is not routinely performed at our center and it is located in a non-endemic region for NPC, and this decision is also influenced by resource limitations.

A retrospective study of 4768 NPC patients revealed neck mass (76%), nasal dysfunction (73%), aural dysfunction (62%), headache (35%), diplopia (11%), facial numbness (8%), weight loss (7%), and trismus (3%) as initial symptoms of presentation [5]. It is said that about half of the patients may have Eustachian tube obstruction causing hearing loss, ear effusion, or fullness. Commonly, cervical lymph nodes enlarge, especially in the posterior triangle and upper jugular level [1]. Another study of 101 NPC patients showed that the most common presenting symptoms were otologic issues (41%), neck mass (39%), nasal symptoms (32%), and headache or cranial neuropathy (16%) [6].

There were few insights of uncommon presentations of NPC where patients presented with fever, trismus, painful neck swellings, visual impairment, ocular pain, triplopia, frequent cold and cough, anorexia, muscle weakness, ptosis, facial pain, bilateral progressive lower limb weakness due to spinal metastasis, headaches, pain, and difficulty in chewing.

It is said that the incidence and distribution of CN involvement in NPC typically include the trigeminal nerve (CN 5) at 38%, the abducens nerve (CN 6) at 26%, and the hypoglossal nerve (CN 12) at 11%, which together account for 75% of all CN palsies. Oculomotor nerve (CN 3) palsies occur in up to 9% of cases. Involvement of the optic (CN 2), trochlear (CN 4), and CN 7 to 11 is much less common, ranging from 1% to 5% [7].

The late-stage presentation of NPC in patients can be attributed to several factors like delayed medical consultation, the ambiguous nature of symptoms that may mislead clinicians, the inherent challenges in conducting a thorough examination of the nasopharynx, and the presence of submucosal lesions that appear normal during clinical assessment [8]. A study revealed that the median time from symptom onset to the first visit to a primary care physician was 6.0 weeks, followed by 2.4 weeks from the primary care visit to an otolaryngology-head and neck surgery(HNS) consultation, 1.1 weeks from the otolaryngology-HNS consultation to a pathological diagnosis, and 5.5 weeks from diagnosis to the start of treatment. A nasopharyngeal lesion was identified in 53% of patients during their first otolaryngology-HNS visit. However, 32% of initial nasal endoscopies and 32% of initial imaging studies failed to detect a nasopharyngeal lesion [6].

Since early-stage NPC symptoms are typically nonspecific, most patients are diagnosed at an advanced stage. Developing an effective primary screening protocol for NPC could, therefore, contribute to earlier detection and improved treatment outcomes. Research indicates that latent EBV infection in nasopharyngeal epithelial cells is crucial for NPC pathogenesis, as EBV normally induces a lytic infection. In a study aimed at developing a reliable method for NPC risk assessment, researchers evaluated the effectiveness of a trans-oral brushing system. This system was designed to be noninvasive, sensitive, and specific, making it suitable for routine, large-scale screening in high-risk populations. The study involved 600 Chinese patients, where a single-use trans-oral brush was employed to rapidly and non-traumatically collect nasopharyngeal epithelial cells for DNA analysis by quantitative polymerase chain reaction (q-PCR) [9].

EBV-related serologic tests are increasingly recognized as valuable tools for the early detection of NPC, particularly in high-risk populations. Although current screening methods in clinics are not fully satisfactory, ongoing research into molecular biomarkers and the use of narrow-band imaging (NBI) holds promise for improving early diagnosis, especially in recurrent mucosal lesions [10].

A large-scale screening involving 56,584 individuals aged 30 and older demonstrated that both the immunoenzymatic method and EBV antibody screening are simple, sensitive, and specific, effectively identifying early NPC cases that might otherwise go unnoticed [11]. Another study says that the most accurate diagnostic approach among EBV-related antibody assays is a three-marker combination of viral capsid antigen (VCA) - immunoglobulin (Ig) A, early antigen (EA) - IgA, and replication and transcription activator (Rta) - IgG. However, from a cost-benefit perspective, a combination of VCA-IgA with either EA-IgA or Rta-IgG could serve as the preferred serodiagnostic strategy for NPC screening and early diagnosis [12]. Evidence supporting the effectiveness of EBV-directed screening in reducing NPC mortality is growing, particularly in regions where familial aggregation is prevalent. Individuals with a family history of NPC exhibit significantly higher EBV positivity rates and an elevated risk of developing the disease, underscoring the potential of familial screening in these endemic areas [13].

Plasma EBV DNA has emerged as a promising biomarker for various clinical applications, including screening, prognostication, treatment planning, and post-treatment surveillance. Despite its potential, comprehensive guidelines and methodological consistency are still needed for global implementation. Recent studies suggest that EBV-based screening is cost-effective in high-incidence populations and could be extended to individuals as young as 40 in select regions [14].

Additionally, pretreatment factors such as age and serum lactate dehydrogenase (LDH) levels have been identified as independent predictors for early-stage NPC, aiding in early detection and more tailored treatment strategies [15]. In clinical settings, the use of convolutional neural networks (CNNs) in conjunction with NBI has shown promise in rapidly distinguishing NPC from benign nasopharyngeal lesions, potentially reducing the need for invasive biopsies and enhancing early diagnostic accuracy [16].

These developments may collectively contribute to more effective early diagnosis and improved outcomes in NPC management.

Treatment of NPC varies by stage: stage I typically involves radiation therapy to the tumor and neck lymph nodes, while stages II, III, and IVA may require combined radiation and chemotherapy, with surgery considered only in recurrent cases. Pretherapy circulating EBV DNA load is an independent prognostic factor. High pre-treatment EBV DNA levels, indicating greater tumor burden, are linked to advanced disease stages and reduced survival [17]. The quantification of plasma EBV DNA has been shown to be useful for monitoring NPC patients and predicting treatment outcomes [18]. Additionally, research has demonstrated that post-radiotherapy plasma EBV DNA levels are significantly associated with increased risks of locoregional failure, distant metastasis, and mortality [19].

As a final point analyzing EBV serology along with an assessment of risk factors in relation to NPC and recognizing clinical presentations such as persistent headaches accompanied by trigeminal neuralgia could be crucial for early diagnosis, prognosis, and anticipating recurrence. Integrating EBV serology into protocols may enhance the identification and suspicion of NPC in high-risk patients, potentially improving outcomes through earlier intervention. However, further research may be needed to validate its use more extensively and establish its scientific reliability in broader clinical practice.

## Conclusions

NPC usually presents as neck mass, nasal dysfunction, and aural dysfunction, along with less frequent symptoms like headache and CN palsies. Unusual presentations, like muscle weakness and trismus, underscore the diagnostic challenges posed by NPC. Effective treatment primarily involves radiotherapy and chemotherapy, with surgery reserved only for recurrent cases without intracranial extension.

The importance of early diagnosis is highlighted by two case reports, one featuring persistent headache misdiagnosed as migraine with associated giddiness due to VIII nerve involvement and another with severe facial pain and inconclusive initial biopsy results. Both cases underscore the need for clinical awareness of uncommon NPC presentations to avoid delayed diagnosis and treatment and highlight that trigeminal neuralgia may be a presenting symptom of NPC with evidence suggesting that the trigeminal nerve is the most frequently affected CN in NPC. Recognizing these uncommon symptoms can facilitate prompt intervention, improving overall prognosis even in advanced stages. Further research may be needed to develop protocols for the early detection of NPC, potentially incorporating EBV serology and diagnostic nasal endoscopy as screening tools for high-risk patients and those with chronic, non-resolving headaches.
